# The role of ^18^F-fluorodeoxyglucose positron emission tomography/computed tomography in the primary staging of rectal cancer

**DOI:** 10.1186/1477-7819-12-26

**Published:** 2014-02-01

**Authors:** Salih Erpulat Ozis, Cigdem Soydal, Cihangir Akyol, Nalan Can, Ozlem Nuriye Kucuk, Cemil Yagcı, Ayhan Bulent Erkek, Mehmet Ayhan Kuzu

**Affiliations:** 1 Department of Surgery, Medical Faculty, Ufuk University, Ankara, Turkey; 2 Department of Nuclear Medicine, Medical Faculty, Ankara University, Ankara, Turkey; 3 Department of Surgery, Medical Faculty, Ankara University, Ankara, Turkey; 4 Department of Nuclear Medicine, Medicana Hospital, Ankara, Turkey; 5 Department of Radiology, Medical Faculty, Ankara University, Ankara, Turkey

**Keywords:** Rectal cancer, ^18^F-flourodeoxyglucose (FDG) positron emission tomography (PET)/computed tomography, Primary staging

## Abstract

**Background:**

In this study we aimed to determine the need for ^18^F-flourodeoxyglucose (FDG) positron emission tomography/computed tomography (PET/CT) in the preoperative staging of rectal carcinoma in our large patient group according to level and location of tumor.

**Method:**

Totally, 97 patients diagnosed with primary rectal adenocarcinoma between May 2009 and July 2011 were included in the study. Preoperative staging was performed by evaluating contrast-enhanced thoracic, abdominal, and pelvic computed tomographies. After staging by conventional methods, all patients underwent an ^18^F-FDG PET/CT. In all cases, the relationship between ^18^F-FDG uptake and gender, tumor height at the anal canal, localization in the rectal wall, plasma carcinoembryonic antigen levels, histopathological tumor type, and tumor stage were examined.

**Results:**

While the ceCT was normal in 4 (4%) patients, it was positive for the rectum in 93 (95%), pelvic lymph nodes in 22 (22%), and distant metastases in 14 (14%) (liver (8), lung (8), bone (2), distant lymph nodes (6), and uterus (1)). Using computed tomography, disease stages were determined as stage 0, 1, 2, 3, and 4 in 4, 8, 48, 23, and 14 patients, respectively; ^18^F-FDG PET/CT was normal in two (2%) patients. The mean SUVmax of FDG-positive rectal tumors was calculated as 17.31 ± 9.37. Additionally, 18F-FDG uptake was seen in pelvic lymph nodes in 15 (15%) patients and in distant organs in 24 (24%) patients (liver (9), lung (12), bone (5), distant lymph nodes (11), uterus (1), and sigmoid colon (1)). According to an ^18^F-FDG PET/CT, 2, 7, 47, 20, and 21 patients were staged as stage 0, 1, 2, 3, and 4, respectively. In 14 patients (14.4%), the stage of the disease was either changed, and there was a need to make adjustments to the patient’s treatment strategy (*n* = 10), or the type of operation was changed (*n* = 4). In seven patients (0.7%), findings from ^18^F-FDG PET/CT images did not require any changes of the treatment plan.

**Conclusion:**

F-FDG PET/CT provides new findings in addition to conventional techniques in the staging of primary rectal cancer. These findings could change the patients’ treatment strategies.

## Background

Rectal cancer is a common disease and a significant cause of cancer-related deaths. Accurate staging is extremely important in determining the prognosis and assessing tailored therapy protocols for individual patients [1]. The most useful tumor-related factors in the preoperative staging of rectal cancers include the depth of tumor penetration through the rectal wall, the presence or absence of metastasis to regional lymph nodes, the adjacent organ involvement, and the presence of distant metastases. These factors guide therapeutic decisions with regard to performing local excisions, moving patients directly for radical surgery, offering neoadjuvant (chemo)-radiotherapy, or suggesting palliative measures [2-4]. Clinicians have a variety of diagnostic tools to delineate the aforementioned tumor-related factors. Of the available technologies, computed tomography (CT), magnetic resonance imaging (MRI), and endorectal ultrasound (ERUS) have evolved as the best modalities for accurately staging rectal cancer. In addition, fluorine-18 fluorodeoxyglucose positron emission tomography combined with computed tomography (PET/CT) is widely used not only for preoperative staging, but also for assessing the oncologic outcomes of rectal carcinoma [5-8]. Although several studies reported that the addition of ^18^F-FDG PET/CT to conventional imaging methods might alter the treatment algorithm [9], in a recent study, Cipe et al. concluded that routine use of ^18^F-FDG PET/CT for preoperative staging does not impact the disease management of 96.8% of patients [10]. In this study, we aimed to determine the need for ^18^F-FDG PET/CT in preoperative staging of rectal carcinoma according to level and location of tumors in our large patient group.

## Method

### Patients

The patients in this study were recruited from a population of patients who were referred to Ankara University, Department of Surgery, Ankara, Turkey, and Ufuk University Department of Surgery, Ankara, Turkey, between May 2009 and July 2011. Written informed consent was obtained from the patients, and the study was approved by the second Ankara clinical research ethics committee. A total of 97 patients diagnosed with primary rectal cancer were included in this study; 59 patients were male and 38 female. The mean age of the patients was 59.61 years (SD, 13.18 years; age range, 27–84 years). The diagnosis of the patients was made histopathologically by endoscopic biopsy.

After clinical examination and routine laboratory tests, all patients underwent contrast-enhanced thoracic-abdominal-pelvic CT and whole-body ^18^F-FDG PET/CT. Pelvic MRI and ERUS were performed in some cases, as deemed necessary.

### Contrast-enhanced computed tomography (CT)

For the computed tomography examination, a water-soluble contrast agent (76% urographin 100 ml in 1,500 ml water) was administered orally to all patients, and 5-mm-thick images displaying the jugular notch (up to the pubis) were obtained. A radiopaque (Optiray or Visipaque, 120 ml) substance was administered intravenously, and image capturing was repeated. The thorax, entire abdomen, and pelvis were evaluated for the presence of a primary tumor, regional or intra-abdominal lymph nodes, and distant metastases.

### ^18^F-FDG PET/CT

Patients were required to fast for at least 6 h before scanning, and blood glucose levels were checked prior to ^18^F-FDG FDG injection. An oral contrast agent was applied to all patients. Whole-body ^18^F-FDG PET/CT imaging was performed approximately 1 h after an intravenous injection of 296–370 MBq ^18^F-FDG. Patients rested in a quiet room without the administration of a muscle relaxant during the waiting period. Images were obtained from the skull base to mid-thighs while patients were in the supine position. A CT image was obtained from the integrated PET/CT scanner (General Electric, Milwaukee, WI, USA) with the use of a standardized protocol involving 140 kV, 70 mA, a tube rotation time of 0.5 s per rotation, pitch of 6, and section thickness of 5 mm. Immediately after taking the CT images, PET images were acquired for 4 min per bed position. Emission PET images were reconstructed with non-contrast CT data for attenuation correction. Patients were allowed to breathe normally during the procedure. Dual-time PET/CT images were obtained 2 h after FDG injection in patients whose findings were equivocal, such as low ^18^F-FDG uptake in the lymph nodes or ^18^F-FDG uptake in bowel segments without an increase in wall thickness.

Whole-body PET/CT images were interpreted by two experienced nuclear medicine specialists with the goal of reaching a consensus. Comparisons were made between focus, showing an increased uptake, and background/blood pool activity; anatomic confirmation was done using CT images. The criterion for malignancy was accepted as ^18^F-FDG hypermetabolism at the site of pathological changes noted on the CT or via marked focal hypermetabolism at the physiological uptake sites. Maximum standardized uptake values (SUVmax) were calculated for all pathological lesions. Because ^18^F-FDG PET/CT could not give enough information about T staging, ceCT images were accepted as standard for T staging for TNM staging classification of PET/CT images.

The relationship between the ^18^F-FDG uptake and gender, tumor height at the anal canal, position in the rectal wall, change in terms of plasma CEA levels, and type of tumor histopathology was examined.

### Data analysis

Both radiologists and nuclear medicine specialists who were also blinded to the clinical examinations and imaging studies re-examined the patients’ scans. Thoraco-abdomino-pelvic ceCT and ^18^F-FDG PET/CT data were recorded by the research resident, and patients were treated according to their clinically accepted stage. Patients were then followed up so that discordant or incidental findings could be verified by intraoperative examination, imaging, or histology where possible.

### Statistical analysis

Statistical analysis was performed using an SPSS software package (version 15.0, Chicago, IL, USA). The McNemar test, chi-square test, Student’s *t*-test, and Kruskal-Wallis variant analysis were used for measurements and comparisons. The confidence interval was accepted to be 95%.

## Results

This prospective study included 97 consecutive primary rectal cancer patients diagnosed with biopsy. Demographic and disease characteristics of the patients are summarized in Table 1.

**Table 1:** Basic characteristics of the patients in the study

**Total number of patients**	**97**
Gender	
Male	59 (60.8%)
Female	38 (39.2%)
Age (years)	
Male	59.3
Female	60.1
Mean (age range)	60.5 (27–84)
Level of the tumor	
Lower rectum (1–5 cm)	55 (56.7%)
Mid rectum (6–10 cm)	29 (29.9%)
Upper rectum (11–15 cm)	13 (13.4%)
Localization of the tumor in the rectal wall	
Posterior	15 (15.46%)
Anterior	10 (10.31%)
Lateral	37 (38.15%)
Circumferential	35 (36.08%)
Histopathological evaluation of the tumor	
Well differentiated	71 (73.8%)
Poorly differentiated and mucinous	26 (26.2%)
Patients’ serum CEA levels	
Normal (≤5 ng/ml)	50.6%
High (>5 ng/ml)	49.4%

While ceCT was totally normal in 4 (4%) patients, rectal tumors were found in 93 (95%) patients, pelvic lymph nodes in 22 (22%) patients, and distant metastases in 14 (14%) patients (liver (8), lung (8), bone (2), distant lymph nodes (6), and uterus (1)). Disease stages were determined by computed tomography as stage 0, 1, 2, 3, and 4 in 4, 8, 48, 23, and 14 patients, respectively.

^18^F-FDG PET/CT was normal in two (2%) patients. Histopathologies of two FDG negative tumors were mucinous and well-differentiated adenocarcinoma. Mean SUVmax of FDG-positive rectal tumors was calculated as 17.31 ± 9.37. Mean SUVmax of primary tumors of poorly differentiated, well-differentiated adenocarcinoma, and mucinous adenocarcinoma patients were calculated as 18.6 ± 10.1, 15.7 ± 8.2, and 13.5 ± 7.5. The difference between groups was not statistically significant (*p* > 0.05).

Additionally, ^18^F-FDG uptake was seen in pelvic lymph nodes in 15 (15%) patients and in distant organs in 24 (24%) patients (liver (9), lung (11), bone (5), distant lymph nodes (12), uterus (1), and sigmoid colon (1)). Mean SUVmax of metastatic and non-metastatic lymph nodes was calculated as 2.23 ± 1.9 and 0.4 ± 1.0, respectively (*p* > 0.05). Dual time imaging was performed in seven patients with suspected pelvic lymph nodes with low ^18^F-FDG uptake and two patients with bowel uptake. Because of the increase in SUVmax of the suspected pelvic lymph nodes, all of them were accepted as metastatic. Although one out of two bowel uptakes disappeared in dual time imaging, the second one was stable. For this reason, the first one was accepted as physiological bowel uptake and the latter as pathological. According to ^18^F-FDG PET/CT, 2, 7, 47, 20, and 21 patients were staged as stage 0, 1, 2, 3, and 4, respectively. The disease stages, based on computed tomography and ^18^F-FDG PET/CT, are summarized in Table 2.

**Table 2:** Disease stage according to computed tomography and PET/CT

	**Stage**	**TNM**	**No. of patients**			**Stage**	**TNM**	**No. of patients**	
**ceCT**	**0**	**T0N0M0**	4	4	**PET/CT**	**0**	**T0N0M0**	2	2
	**I**	**T2N0M0**	8	8		**I**	**T2N0M0**	7	7
	**II**	**T3N0M0**	48	48		**II**	**T3N0M0**	47	47
	**III**	**T2N1M0**	2	23		**III**	**T2N1M0**	2	20
		**T3N1M0**	11				**T3N1M0**	10	
		**T3N2M0**	9				**T3N2M0**	7	
		**T4N2M0**	1				**T4N2M0**	1	
	**IV**	**T3N0M1**	1	14		**IV**	**T3N0M1**	2	21
		**T3N1M1**	6				**T3N1M1**	7	
		**T3N2M1**	3				**T3N2M1**	5	
		**T4N1M1**	4				**T4N1M1**	7	

**Figure 1:**
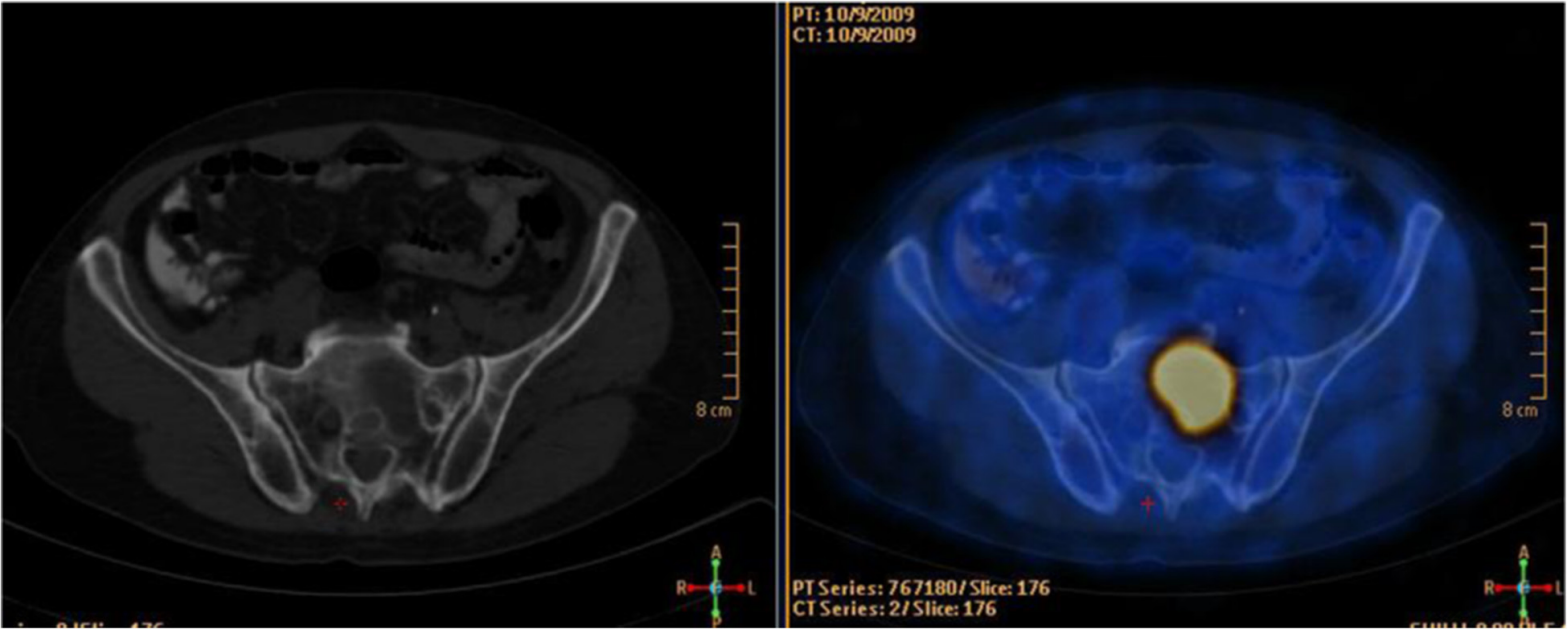
**Transaxial CT and fused PET/CT images of a 65-year-old male patient with rectal cancer.**^18^F-FDG PET/CT showed intense ^18^F-FDG uptake (SUVmax: 11.3) in sacral metastases, which could be easily missed by ceCT.

**Figure 2:**
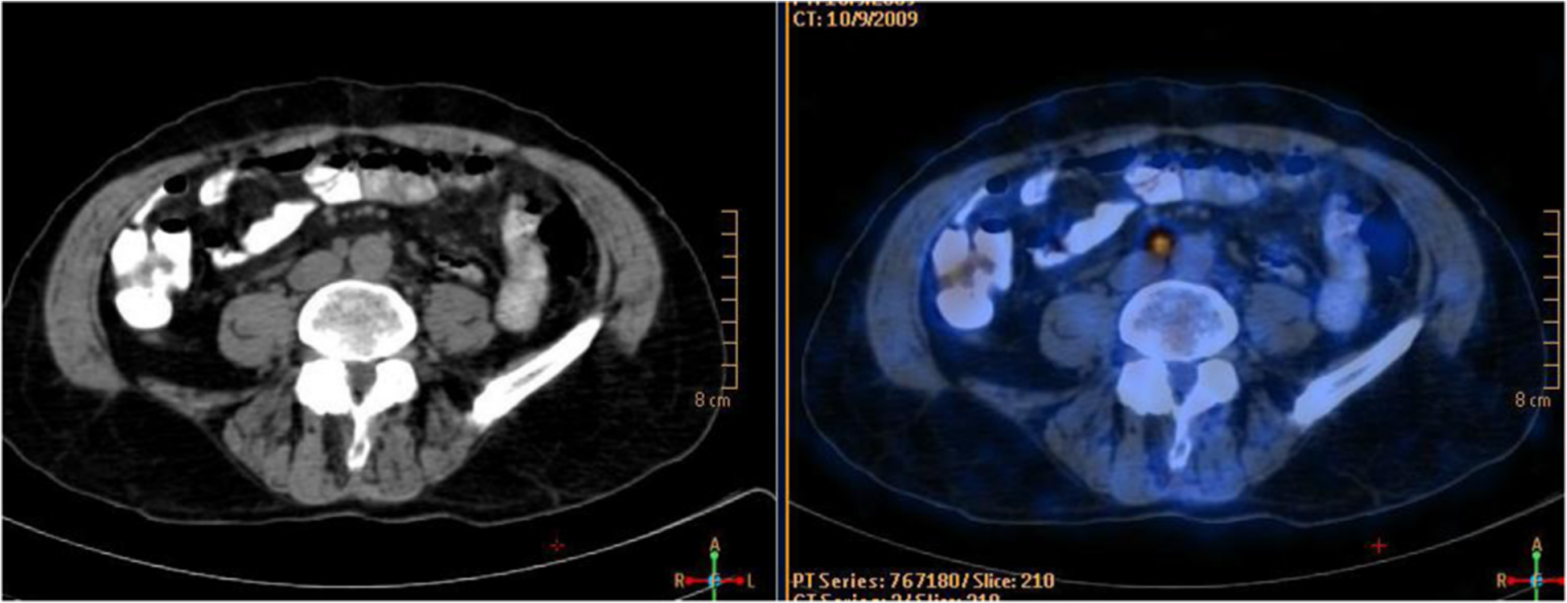
**Transaxial CT and fused PET/CT images of the same patient.**^18^F-FDG PET/CT showed intense ^18^F-FDG uptake (SUVmax: 5.2) in millimeter-sized aortocaval lymph nodes which were not reported as pathological on ceCT.

ceCT and ^18^F-FDG PET/CT were compatible in 67 (72%) patients. In addition to the data obtained using conventional methods, ^18^F-FDG PET/CT provided additional data (*p* < 0.01) for 21 (21.6%) out of 97 patients (19%). Specifically, ^18^F-FDG PET/CT detected more distant organ metastases (liver (1), bone (3), lung (4), and distant lymph nodes (5)). In two patients, the primary rectal tumor focus could not be seen using ^18^F-FDG PET/CT, while CECT was useful for finding the tumor location. In 14 patients (14.4%), the stage of the disease changed, and there was a need to make adjustments to the patient’s treatment strategy (*n* = 10) or operation type changed (*n* = 4). Changes in stage are demonstrated in Table 3. In seven patients (0.7%), the findings from ^18^F-FDG PET/CT images did not require any changes in the treatment plan. Two examples are presented in Figures 1 and 2.

**Table 3:** Change in stage according to PET/CT

**From**	**To**	**No. of patients**
Stage 0	Stage 1	2
Stage 1	Stage 3	2
Stage 1	Stage 4	1
Stage 2	Stage 4	1
Stage 3	Stage 4	5
Stage 4	Stage 3	3

In all cases, the relationship between the compatibility of ^18^F-FDG PET/CT and ceCT with sex, tumor height at the anal canal, localization at the rectal wall, serum CEA level, tumor histopathological type, and stage of tumor was examined, with no statistically significant relationships observed.

## Discussion

In consideration of the higher incidence and disease-related death rates, accurate preoperative staging of rectal cancer is crucial. The staging must be reliable in order to select the appropriate treatment for patients. Treatment options alter according to preoperative stage. Surgery is the main curative treatment for local disease with/without limited liver metastases. Distant metastases from planned operation sites could affect the treatment strategy. The treatment of rectal cancer, especially in patients with a high risk of recurrence, has shifted from purely surgical treatment to multimodal therapy with neoadjuvant treatment [4]. The purpose of preoperative staging is to evaluate the depth of the tumor’s local infiltration, degree of lymph node involvement, and presence of distant organ metastases. Conventionally, thoraco-abdomino-pelvic ceCT and pelvic MRI have been the choice of modalities for preoperative staging of rectal cancer. ceCT and MRI could provide information about the location, size, and local invasion of the primary tumor and also enlargement of regional lymph nodes. However, they have limited value during the evaluation of regional lymph node involvement because a normal-sized lymph node may have a tumor, and an enlarged lymph node may be reactive [11].

^18^F-FDG PET can show the metabolic activity of malignant tumors and is accepted as an important imaging method in the diagnosis and staging of many malignant diseases [12-18]. ^18^F-FDG PET allows for better staging of many cancers, such as esophageal and non-small cell lung cancer, and thus has contributed to an improvement in the treatment of patients [19]. Some studies have reported that ^18^F-FDG PET imaging is effective in the evaluation of patients with suspected recurrent colorectal cancer [20-22] and patients with local advanced rectal cancer [23]. Regarding primary rectal cancer studies, it has been reported that ^18^F-FDG PET imaging leads to changes in the cancer stage in one-third of patients [24]. As a result of advances in the industry, hybrid devices have been designed to allow simultaneous imaging and interpretation of both anatomical (CT) and functional (^18^F-FDG PET) images [25]. ^18^F-FDG PET/CT imaging was shown to be more effective in patients with locally recurring and metastatic colorectal cancer [26].

The role of ^18^F-FDG PET/CT in the primary staging of rectal cancer is controversial. First, ^18^F-FDG PET/CT could not give enough information about the T stage because of non-contrast-enhanced and low-dose CT images. Additionally, it has a limited role in the evaluation of millimeter-sized lung nodules and liver lesions because of the low spatial resolution. However, it has some advantages over conventional methods, especially in the evaluation normal-sized regional lymph nodes, nonspecified liver lesions, and small bone metastases. For these reasons, like in our study, the need for and additional role of ^18^F-FDG PET/CT for the primary staging of rectal cancer have been subjects of focus [19,27].

Further, in 21.6% of the cases in our study, ^18^F-FDGPET/CT provided additional findings other than those previously observed by conventional tomography (*p* < 0.01). These new findings included metastases in the liver, lung, bone, and lymph nodes, invasions of adjacent organs, and synchronous tumors identified in the colon. The disease was upstaged in 11 cases. ^18^F-FDG uptake was detected two patients’ primary tumorsthose were normal in ceCT, local lymph node involvement was detected in normal-sized lymph nodes, ^18^F-FDG uptake was seen in suspected liver lesions, lung nodules, and distant lymph nodes, and there were bone metastases that were not detected by ceCT. Additionally, the disease was down staged in three patients whose suspected liver lesions and lung nodules were not ^18^F-FDG avid. In a total of 14 patients there was a need to change the treatment strategy or change the surgical intervention. Gearhart et al. [27] investigated the role of 18F-FDG PET⁄CT in the initial staging of rectal cancer and reported their results. They studied 37 patients for staging of rectal cancer and reported discordant findings in 38%, while 10 of the 37 patients (27%) underwent a change in stage after ^18^F-FDG PET⁄CT. Eglinton et al. [28] reported their results in 20 rectal cancer patients and found that ^18^F-FDG PET/CT detected discordant or incidental findings in about half of the patients, which in turn led to a change in staging in 30% of them. However, they did not observe significant results that impacted treatment decisions. Bassi et al. [18] reported that ^18^F-FDG PET/CT imaging changed staging in 16% of the patients and significantly increased the target volume in contouring radiotherapy. Recently, Davey et al. [19] evaluated the role of ^18^F-FDG PET⁄CT in 86 patients and reported a 31% change in stage due to ^18^F-FDG PET⁄CT. On the contrary, some studies advocate that preoperative staging is not necessary in primary rectal cancers [10]. For instance, Kwak et al. [6] reported that preoperative ^18^F-FDG PET/CT and CT showed similar results in determining lymph node metastases.

In our patients, ^18^F-FDG PET/CT did not show primary rectal tumors in two patients. These patients showed histopathological subtypes of tumor that were mucinous and well-differentiated adenocarcinoma. Mucinous contentx and well-differentiated tumors are factors that decrease ^18^F-FDG uptake and could be the cause of false-negative results. Also, ^18^F-FDG PET/CT could not provide information about the degree of infiltration of the rectal wall because of the limited spatial resolution of the study. For this reason, ^18^F-FDG PET/CT is not recommended for T staging. In the future, contrast-enhanced ^18^F-FDG PET/CT examinations could solve this problem and could eliminate the need for ceCT imaging in a separate course. However, these false-negative results did not change the treatment management of these patients because primary rectal tumors had already been detected by biopsy.

In this study, in addition to comparing conventional tomography with ^18^F-FDG PET/CT, we also evaluated the serum CEA levels, localization of the tumor in the rectal wall (posterior, anterior, lateral, and all around), endoscopic assessment of the distance of the tumor from the anal canal (1–5 cm lower rectum, 6–10 cm mid-rectum, 11–15 upper rectum), and the degree of histopathological differentiation of the tumor. Moreover, we assessed the statistical relationship of the above with the compatibility of ceCT and ^18^F-FDG PET/CT, and, to that end, we did not find any relationship. Additional information obtained from ^18^F-FDG PET/CT seems to be independent from these factors.

We could not evaluate the sensitivity and specificity of 18F-FDG PET/CT because of the lack of histopathological confirmation of all FDG-avid lesions. In this study, we aimed to describe the need for and additional role of PET/CT in the staging and clinical management of our patient group.

## Conclusion

According to our results, ^18^F-FDG PET/CT has contributed positively to the staging of primary rectal cancer patients and led to changes in the treatment strategy of 14.4% of the patients. It seems to be complementary to ceCT in patients with suspected findings. This conclusion was reached by one of the largest studies in the current literature. Thus, we recommend ^18^F-FDG PET/CT as one of the methods to be consulted in the staging of patients with primary rectal cancer.

## Competing interests

The authors declare that they have no competing interests.

## Authors’ contributions

SEO, CA, ABE and MAK selected and informed patients to recruit to the study and operated the patient, CS, NC and ONK evaluated 18F-FDG PET/CT images, CY assessed ceCT images, SEO documented the data and CS did statistical analysis, SEO, CS, MAK and ONK wrote the manuscript. All authors read and approved the final manuscript.
